# Establishment of a continuous untransfected human corneal endothelial cell line and its biocompatibility to denuded amniotic membrane

**Published:** 2011-02-15

**Authors:** Tingjun Fan, Jun Zhao, Xiya Ma, Xiaohui Xu, Wenzhuo Zhao, Bin Xu

**Affiliations:** Key Laboratory for Corneal Tissue Engineering, Ocean University of China, Qingdao,China

## Abstract

**Purpose:**

To establish an untransfected human corneal endothelial (HCE) cell line and characterize its biocompatibility to denuded amniotic membrane (dAM).

**Methods:**

Primary culture was initiated with a pure population of HCE cells in DMEM/F12 media (pH 7.2) containing 20% fetal bovine serum and various supplements. The established cell line was characterized by growth property, chromosome analysis, morphology recovery, tumorigenicity assay, and expression of marker proteins, cell-junction proteins, and membrane transport proteins. The biocompatibility of HCE cells to dAM was evaluated by light microscopy, alizarin red staining, immunofluorescence assay, and electron microscopy.

**Results:**

HCE cells proliferated to confluence 6 weeks later in primary culture and have been subcultured to passage 224 so far. A continuous untransfected HCE cell line with a population doubling time of 26.20 h at passage 101 has been established. Results of chromosome analysis, morphology, combined with the results of expression of marker protein, cell-junction protein and membrane transport protein, suggested that the cells retained HCE cell properties and potencies to form cell junctions and perform membrane transport. Furthermore, HCE cells, without any tumorigenicity, could form confluent cell sheets on dAMs. The single layer sheets that attached tightly to dAMs had similar morphology and structure to those of HCE in situ and had an average cell density of 3,413±111 cells/mm^2^.

**Conclusions:**

An untransfected and non-tumorigenic HCE cell line has been established, and the cells maintained positive expression of marker proteins, cell-junction proteins and membrane transport proteins. The cell line, with excellent biocompatibility to dAM, might be used for in vitro reconstruction of HCE and provides a promising method for the treatment of diseases caused by corneal endothelial disorders.

## Introduction

The human corneal endothelium (HCE) is the single layer of cells, located at the posterior end of the cornea between the stromal layer and the aqueous humor, that is critically involved in maintaining corneal thickness or transparency [1]. The density of HCE cells decreases with age [2], disease [3], intraocular surgery [4], or laser procedures [5]. Repair of mature HCE monolayer in response to cell loss occurs mainly by cell enlargement and migration [6]. Although adult HCE cells have lost their proliferative activity, arrested in G_1_-phase in vivo, and are generally difficult to be cultured in long-term, they do retain proliferative capacity [1,7].

HCE cell lines could provide efficient models for studies of cellular specification, cellular signaling, cell replacement, in vitro reconstruction of tissue-engineered HCE (TE-HCE), immunology of HCE graft rejection, and molecular pathways regulating normal HCE cell homeostasis [7,8]. The main difficulties encountered in establishing HCE cell lines are maintenance of morphological differentiation and functional status, induction of their proliferation, and prevention of keratocyte/fibroblast overgrowth [9]. Although numerous attempts have been made to cultivate HCE cells for protracted periods in vitro [7,8,10,11], cultured HCE cell lines have only been developed by transfection with viral oncogenes coding for Ha-ras, SV40 large T antigen and HPV16 E6/E7 [12-15]. The effectiveness of these transfected cell lines as potential research models has been hampered by genetic instability, abnormal phenotypes, and tumorigenicity, precluding their effective use in studies of normal endothelial cell biology and clinical corneal endothelial cell replacement [16]. No untransfected HCE cell line has been established before this study, except for the two untransfected rabbit corneal endothelial cell lines that we established previously [16,17].

Since no definitive markers for HCE cells have been identified, HCE cells can only be characterized with various marker proteins such as neuron specific enolase (NSE), type IV collagen, and vascular endothelial growth factor receptor II (FLK-1), various cell-junction proteins such as zonula occludens protein 1 (ZO-1), N-cadherin, connexin 43 and integrin αv/β5, and various membrane transport proteins such as aquaporin 1 (AQP1), Na^+^/K^+^-ATPase, voltage-dependent anion channels (VDACs), chloride channel proteins (ClCs), sodium bicarbonate cotransporter 4 (NBC1), and cystic fibrosis transmembrane conductance regulator (CFTR) [9,12-18]. To provide a viable model for studying HCE cells and reconstruction of TE-HCE for clinical HCE replacement, the present study was intended to establish a continuous untransfected HCE cell line, characterize its intrinsic property and its biocompatibility to denuded amniotic membranes (dAMs).

## Methods

### Animals and materials

Corneas from a woman (26 years old) who died due to cerebral hemorrhage were obtained from The Affiliated Hospital of Medical College, Qingdao University (Qingdao, China), with permission from her next of kin. The usage of the corneas as the source of HCE cells for in vitro culture was approved by the Medical Ethics Committee of the hospital and the privacy of the patient was protected in compliance with the Declaration of Helsinki. SPF BalB/c nude mice (male), weighing 18 to 22 g, were purchased from Slaccas Experimental Animal Co., Ltd. (Shanghai, China). All animals were treated in accordance with the ARVO Statement for usage in tumorigenesis assay, and were approved by the Clinical Research Ethics Committee of New Drug Evaluation Center, Shandong University (Jinan, China).

Fresh amniotic membranes (AMs), stored in −20 °C glycerol, were obtained from Shandong Eye Institute of Shandong Medical Academy. The AMs were washed twice with 0.9% physiologic saline and disinfected with 1,000 IU/ml tobramycin for 20 min. After a further wash with physiologic saline, the AMs, with the epithelial surface down, were spread uniformly without folds on filter papers fully immersed in 0.02% EDTA-0.25% trypsin (1:1; Sigma-Aldrich, St. Louis, MO) solution. After digestion at 37 °C for 30 min, AMs were washed and their epithelial surfaces were scraped gently with a sterile cell-scraper to obtain denuded AMs (dAMs). Human corneal stromal (HCS) cells, from an HCS cell line established in our laboratory, were cultured in Dulbecco’s Modified Eagle’s Medium/Ham’s Nutrient Mixture F12 (DMEM/F12, 1:1) medium (pH 7.2; Invitrogen, Carlsbad, CA) containing 20% bovine calf serum (BCS; Invitrogen) at 37 °C with 5% CO_2_. During logarithmic phase, the culture supernatant of HCS cells was collected and centrifuged at 2,000× g for 30 min.

### In vitro culture of HCE cells

In vitro culture of HCE cells was initiated as described previously [16]. Donated fresh human corneas were placed flatly in a 35 mm culture dish. About 0.5 ml of 0.25% trypsin was infused into their hollows for digestion of about 2 min. The trypsin solution was discarded and the corneas were rinsed with 10% BCS-DMEM/F12 medium (pH 7.2; Invitrogen) and cut into quarters. The quartered pieces were attached directly onto a 0.05% gelatin (Sigma-Aldrich)-coated wells of a 24 well culture plate with their endothelial surface down, and cultured in 10% fetal bovine serum (FBS)-DMEM/F12 medium (Invitrogen) at 37 °C with 5% CO_2_. The corneal pieces were detached and re-attached sequentially into new gelatin-coated wells for a time interval of 36 h, 24 h, 12 h and 6 h, respectively. The wells containing only a pure population of HCE cells were selected, and the cells in these wells were collected. The pure HCE cells were resuspended in 1 ml of 20% FBS-containing DMEM/F12 medium with antibiotics, and split into two new gelatin-coated wells and cultured at 37 °C with 5% CO_2_. Twenty-four h later, the culture medium was further supplemented with 10 ng/ml basic fibroblast growth factor (bFGF), 10 ng/ml epidermal growth factor (EGF), 50 μg/ml N-acetylglucosamine hydrochloride, 50 μg/ml glucosamine hydrochloride, 0.8 mg/ml chondroitin sulfate, 50 μg/ml oxidation-degradation products of chondroitin sulfate (all obtained Sigma-Aldrich), 50 μg/ml carboxymethyl-chitosan (AK Scientific, Mountain, CA), 5 ng/ml bovine ocular extracts (Bosen Biologic Pharmaceutical Inc., Xi’an, China), and 10% (v/v) culture supernatant of HCS cells at logarithmic phase. The primary culture was initiated at 37 °C with 5% CO_2_ with medium refreshed at 5 day intervals. Once a confluent monolayer had formed, HCE cells were subcultured with 0.25% trypsin as described previously [17]. Briefly, the trypsin-digested HCE cells were suspended and inoculated into new culture wells at a ratio of 1:2, and the same method was used during subsequent subcultures of HCE cells. From passage 30, the cells were subcultured in 20% FBS-containing DMEM/F12 medium without growth factors and the other supplements as described above.

### Growth properties

Passage 101 HCE cells were harvested and suspended in 20% FBS-DMEM/F12 medium to a density of 2.0×10^5^ cell/ml, and the growth properties of the cells were measured as previously described [17]. Briefly, the number of HCE cells in each well was counted at a 12 h interval, and the average value of 3 wells was used to plot their growth curve. Data shown are the mean±SD of three experiments (n=3), and the mean value of cell density was used to protract the growth curve and calculate the population doubling time of HCE cells.

### Chromosome analysis

The chromosome specimens of the passage 101 HCE cells at logarithmic phase were prepared as previously described [17]. Briefly, harvested HCE cells were suspended in 0.3% KCl hypotonic solution and fixed with Carnoy’s solution, and chromosome specimens were stained with Giemsa after being air dried. Chromosome numbers of 300 metaphase cells were counted under an E200 microscope (Nikon, Tokyo, Japan) and statistically analyzed.

### Morphology recovery

Morphology recovery of HCE cells at passage 101 was performed with 5%, 10%, 15% BCS-supplemented MEM, DMEM and DMEM/F12 medium (Invitrogen), respectively, as previously described [17]. Briefly, the HCE cells in different BCS-containing mediums were all cultured at 37 °C with 5% CO_2_. The medium was refreshed at 3-day intervals with the same BCS-containing medium, respectively, and the morphological changes of HCE cells were monitored daily under a Nikon E200 microscope (Nikon, Tokyo, Japan).

### Determination of protein expression by western blot

Total protein was extracted from passage 101 HCE cells, and 18 μl of protein extracts were directly applied to 7.5% sodium dodecylsulfate polyacrylamide gel. Gel samples were electrophoresed and electroblotted onto a 0.45 μm nitrocellulose membrane using an electroblotting system (ATTO, Tokyo, Japan) according to the manufacturer’s instructions. After blocking with 10% BCS (Invitrogen) for 1 h at 37 °C, the blots were probed with mouse anti-human monoclonal antibodies of collagen type IV, von Willebrand factor (vWF), FLK-1, and keratin 12 (1:500; Millipore, Billerica, MA) overnight at 4 °C, respectively. The negative control blots were incubated with 0.05 M Tris-NaCl buffered saline (TBS) (pH7.2). Horseradish peroxidase (HRP)-conjugated goat anti-mouse IgG (1:500; Biosynthesis Biotechnology, Beijing, China) was used as secondary antibodies. The blots were developed with kits of 3,30-diaminobenzidine tetrahydrochloride (DAB) in TBS (pH7.2; Biosynthesis Biotechnology).

### Determination of protein expression by immunocytochemistry

Immunocytochemistry of NSE expression of HCE cells at passage 101 was performed with rabbit anti-human NSE (1:100; Signet Laboratory Inc., Dedham, MA), HRP-conjugated AffiniPure goat anti-rabbit IgG (1:100; Biosynthesis Biotechnology) and DAB kits (Biosynthesis Biotechnology) as previously described  [17]. Briefly, after being fixed with methanol (Sigma-Aldrich), treated with 0.5% Triton X-100 (Sigma-Aldrich) and 3% H_2_O_2_ (Sigma-Aldrich), and blocked with 5% BCS (Invitrogen), the HCE cells cultured on coverslips were in turn incubated with rabbit anti-human NSE and HRP-conjugated goat anti-rabbit IgG, and DAB kit stained specimens were photographed with a Nikon E200 microscope. Omission of primary antibodies was used as negative controls.

Immunofluorescence staining of cell-junction protein expression of HCE cells at passage 101 was performed with goat anti-human zonula occludens protein 1 (ZO-1) polyclonal antibody (1:50; Santa Cruz Biotechnology, Santa Cruz, CA), mouse anti-human N-cadherin monoclonal antibody (1:50; Santa Cruz), mouse anti-human connexin 43 monoclonal antibody (1:250; Chemicon, Temecula, CA), mouse anti-human integrin αv/β5 monoclonal antibody (1:50; Santa Cruz), and fluorescent isothiocyanate (FITC)-conjugated rabbit anti-goat IgG antibody or rhodamine B isothiocyanate (RBITC)-conjugated goat anti-mouse IgG antibody or FITC-conjugated goat anti-mouse IgG antibody (1:100; Biosynthesis Biotechnology) as previously described [16], and visualized with a Nikon Eclipse TE2000-U inverted fluorescent microscope (Nikon, Tokyo, Japan). Briefly, after being fixed with 4% paraformaldehyde (Sigma-Aldrich), treated with 0.5% Triton X-100 (Sigma-Aldrich), and blocked with 5% BCS (Invitrogen), the HCE cells were incubated with ZO-1, N-cadherin, connexin 43, and mouse integrin αv/β5 antibody, respectively, and then incubated with FITC-conjugated rabbit anti-goat IgG or RBITC-conjugated goat anti-mouse IgG or FITC-conjugated goat anti-mouse IgG. Omission of primary antibodies was used as negative controls.

### Determination of mRNA expression by real-time PCR

Real-time PCR was performed according to the methods described by Xiao et al. [19]. Total RNAs of primary cultured and subcultured HCE cells were extracted with NucleoSpin RNAII Assay Kit (Macherey-Nagel, Germany), respectively. The first strand cDNA was synthesized from 2 μg of total RNA in a 20 μl reaction mixture with PrimeScriptTM 1st Strand cDNA Synthesis kit (Takara, Ishiyama, Japan). Real-time PCR of target genes, including human aquaporin 1 (*AQP1*), Na^+^/K^+^-ATPase α1 polypeptide (*ATP1A1*), voltage-dependent anion channel 2 and 3 (*VDAC2*, *VDAC3*), chloride channel protein 2 and 3 (*ClC2*, *ClC3*), sodium bicarbonate cotransporter 4 (*NBC1*), and cystic fibrosis transmembrane conductance regulator (*CFTR*), was performed on the ABI Prism 7500 System (ABI, Foster City, CA). The primer sequences for these reactions were designed on the basis of the published human gene sequences (Table 1). The housekeeping gene of human glyceraldehyde-3-phosphate dehydrogenase (*GAPDH*) was used as an internal control. Quantification of the amplified product was done on a cycle-by-cycle basis via the acquisition of a fluorescent signal generated by binding of the fluorophore SybrGreen I (Roche Diagnostics, Shanghai, China) to double-stranded DNA. Each sample was assayed in duplicate with Taqman Universal PCR Master Mix (Applied Biosystems, Foster City, CA). The cycling conditions were as follows: 10 min at 95 °C and 40 cycles of amplification for 15 s at 95 °C and 1 min at 60 °C. The quantification data were analyzed with the 7500 System Software (Applied Biosystems) according to the manufacturer’s instructions. Data shown are the mean±SD of three experiments.

**Table 1:** Oligonucleotide sequences used in this study.

**Name**	**Sequence**	**Accession**
*AQP1* (human aquaporin 1)	F: 5-TGCCATCGGCCTCTCTGTAG-3	AB451275
	R: 5-AAGGACCGAGCAGGGTTAATC-3	
*ATP1A1* (human Na^+^/K+ ATPase, alpha 1 polypeptide)	F: 5-TCACTCCCCCTCCCACTACTC-3	BC003077
	R: 5-CATTGAGAACCCCCCAAAGA-3	
*VDAC2* (human voltage-dependent anion channel 2)	F: 5-CCTTGGTTGTGATGTTGACTTTGA-3	BC012883
	R: 5-CCAGCCCTCATAACCAAAGACA-3	
*VDAC3* (human voltage-dependent anion channel 3)	F: 5-AGGGTGGCTTGCTGGCTAT-3	BC056870
	R: 5-TTGTAACCCAGGGCGAAATT-3	
*CLCN2* (human chloride channel protein 2)	F: 5-CCTGGCTTTGGACAGTTCATG-3	AF026004
	R: 5-CCACGTCCGATTGTCAAACA-3	
*CLCN3* (human chloride channel protein 3)	F: 5-CAAGTCCACGAAATTTGGAAAGT-3	AF029346
	R: 5-TTAGGGAAGGCTATCACAGCAGTA-3	
*NBC1* (human sodium bicarbonate cotransporter 4)	F: 5-CCTCAGCTCTTCACGGAACTG-3	Q9Y6R1
	R: 5-GCTGTTTCCTTCCACTCCATCT-3	
*CFTR* (human cystic fibrosis transmembrane conductance regulator)	F: 5-GCACGAAGGAGGCAGTCTGT-3	NM_000492
	R: 5-TGCTGTTGTCTTTCGGTGAATG-3	
*GAPDH* (human glyceraldehyde-3-phosphate dehydrogenase)	F: 5-CTGCCCAGAACATCATCCCT-3	EU742868
	R: 5-GGTCCTCAGTGTAGCCCAAGA-3	

Note: F: forward primers; R: reverse primers.

### Tumorigenesis asay

Tumorigenesis assay of the HCE cells at passage 101 was performed as previously described [16]. About 1.0×10^7^ HCE cells at logarithmic phase was inoculated subcutaneously into one of the forehand oxters of each of 10 BalB/c nude mice respectively (n=10), while 10 BalB/c nude mice inoculated with 1.0×10^7^ HeLa cells per mouse were used as positive controls. The tumorigenic status of the inoculated mice was monitored daily. After 60 days, the skin of the oxter of inoculated mice was surgically opened and tumorigenic status was examined.

### Biocompatibility to dAMs

Biocompatibility assay of HCE cells to dAM was performed as previously described [16]. Passage 101 HCE cells at logarithmic phase were collected, and the density of cell suspension was adjusted to 1.67×10^6^ cell/ml with 20% FBS-containing DMEM/F12 medium. Then 1 ml cell suspension was plated into 24 well culture plates coated with dAMs, and cultured at the same conditions as described above. Culture medium was refreshed entirely at 3 day intervals and the morphology and growth status were monitored daily. The morphology of cell sheets formed was examined with a Nikon Eclipse TS100 inverted microscope and a JSM2840 scanning electron microscope (SEM; JEOL, Tokyo, Japan). The cell junction status was evaluated with 1% alizarin red staining and immunocytochemistry (the same method as described above). The continuous monolayer state of HCE cell was examined by freeze section and hematoxylin-eosin (HE) staining, and the attachment status of cell sheets and dAMs were examined with a H700 transmission electron microscope (TEM; Hitachi, Tokyo, Japan).

### Statistical analysis

Data are expressed as mean±SD in triplicates or decuplicates and analyzed for statistical significance with ANOVA single factor.

## Results

### In vitro culture of HCE cells

**Figure 1:**
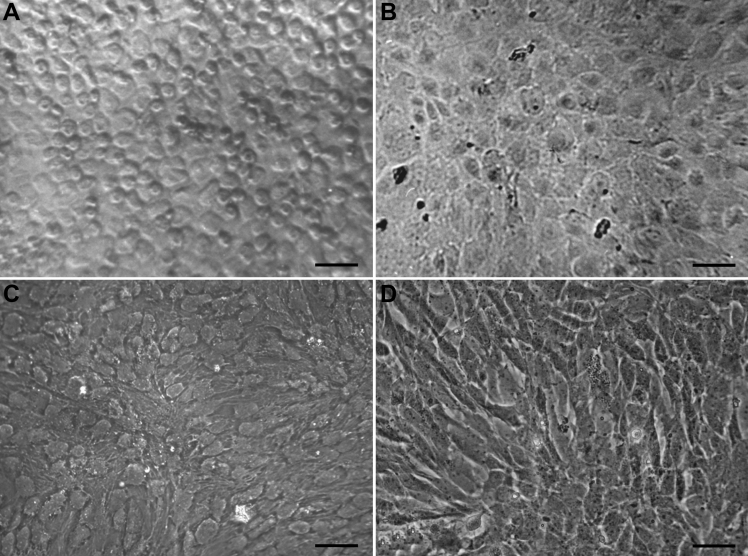
In vitro culture of HCE cells. **A**: HCE cells 36 h after plating, showing the non-spread polygonal cell morphology, i.e., corneal endothelial-like morphology. **B**: The monolayer formed by HCE cells 6 weeks after primary culture initiation, showing the plump polygonal cell morphology. **C**: Passage 101 HCE cells, showing the co-existence of endothelioid and fibroblast-like morphology. **D**: Passage 224 HCE cells, showing the co-existence of elongated polygonal and fibroblast-like morphology. Scale bar: 50 μm.

**Figure 2:**
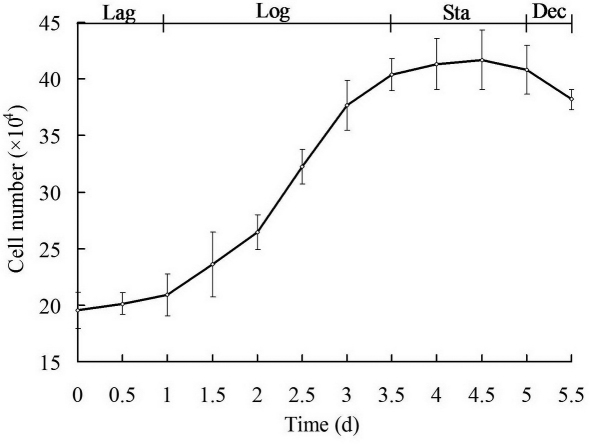
The growth curve of HCE cells at passage 101. The cells had a population doubling time of 26.20 h. The results represent means±SD (n=3). Lag, lag phase; Log, exponential or logarithmic phase; Sta, stationary phase; Dec, decline phase.

Numerous pure HCE cells were found to attach to the bottom of the wells when the corneal pieces were detached 36 h after initiation of attachment (Figure 1A). And these cells, with high transparency, were almost all in non-extended hexagonal or polygonal morphology. After the collected pure HCE cells were cultured in 20% FBS-DMEM/F12 medium with various supplements, the HCE cells grew into a confluent monolayer 6 weeks later (Figure 1B). And the cells were plump and most of them in polygonal morphology. During subsequent subculture, the polygonal morphology of HCE cells began to elongate slightly (Figure 1C). Once subcultured in 20% FBS-DMEM/F12 medium without growth factors and the other supplements, the cells began to elongate into the fibroblast morphology (Figure 1D). After three year’s subculture, a novel continuous untransfected HCE cell line had been established and subcultured to passage 224 by now. The HCE cells grew and proliferated at a steady rate, and their doubling time was calculated to be 26.20 h at passage 101 (Figure 2).

### Chromosome analysis of the HCE cell line

**Figure 3:**
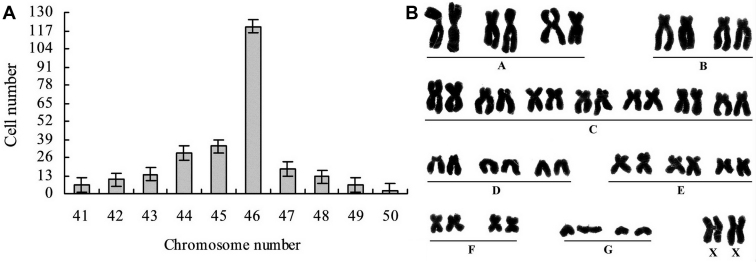
Chromosome analysis of HCE cells at passage 101. **A**: The HCE cells had chromosomal aneuploidy with chromosome numbers ranged from 41 to 50. Among them, HCE cells with chromosome number of 46 were about 47.8%. **B**: Diploid karyotype of a HCE cell with a chromosome number of 46.

Chromosome analysis showed that HCE cells at passage 101 exhibited chromosomal aneuploidy with chromosome numbers ranged from 41 to 50, and the ratio of HCE cells with a chromosome number of 46 is about 47.8% (Figure 3), suggesting that the modal chromosome number of the established HCE cell line was still 46.

### Morphology recovery of HCE cells

**Figure 4:**
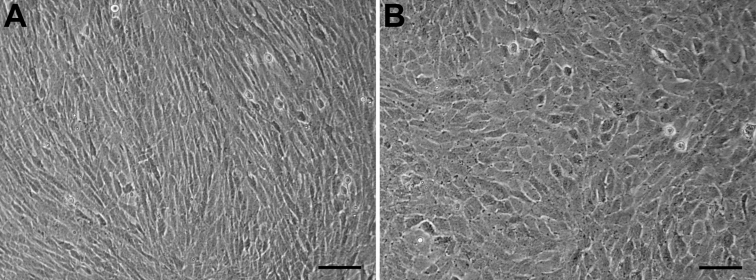
Morphology recovery of HCE cells at passage 101. **A**: Control HCE cells cultured in 20% FBS-containing DMEM/F12 (1:1) medium, showing the fibroblastic morphology. **B**: HCE cells cultured in 5% BCS-containing MEM medium, showing the more corneal endotheloid-like morphology. Scale bar: 50 μm.

After passage 101 HCE cells were cultured in 5%, 10% and 15% BCS-containing MEM, DMEM, and DMEM/F12 medium, respectively, for 2 passages, morphological changes of the cells occurred. The HCE cells cultured in 5% BCS-MEM medium underwent dramatic morphological changes from fibroblastic to polygonal on day 10 (Figure 4), implying that the HCE cell line still had inherent properties of corneal endothelial cells without transdifferentiation.

### Marker protein expression of HCE cells

**Figure 5:**
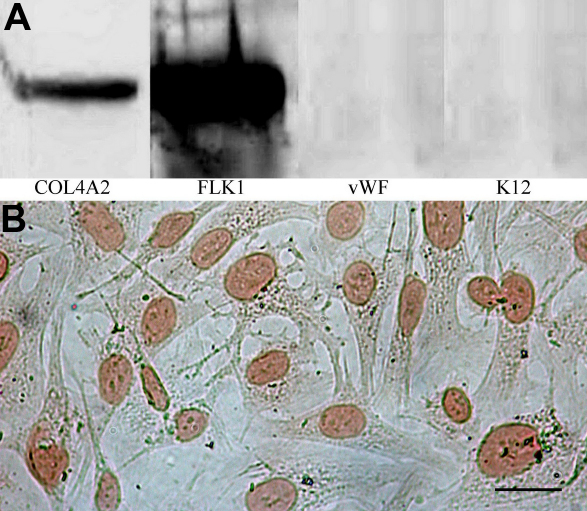
Expression of marker proteins of HCE cells at passage 101. **A**: western blot, showing the positive expression of COL4A2 and FLK1, and negative expression of vWF and keratin 12 of HCE cells. **B**: immunocytochemistry, showing the positive expression of NSE. COL4A2, type IV human collagen α2 polypeptide; FLK1, human vascular endothelial growth factor receptor-2; vWF, human von Willebrand Factor; keratin 12, human keratin 12; NSE, neuron specific enolase.

Results of marker protein expression are shown in Figure 5. Results of western blot revealed that passage 101 HCE cells maintained positive expression of type IV human collagen α2 polypeptide (COL4A2) and human vascular endothelial growth factor receptor 2 (FLK1), but not human von Willebrand Factor (vWF) and human keratin 12 (Figure 5A). Results of immunocytochemistry showed that the HCE cells maintained positive expression of NSE (Figure 5B). All these suggested that the cells of the established cell line had properties of HCE cells, not vascular endothelial cells or corneal epithelial cells.

### Cell-junction protein expression of HCE cells

**Figure 6:**
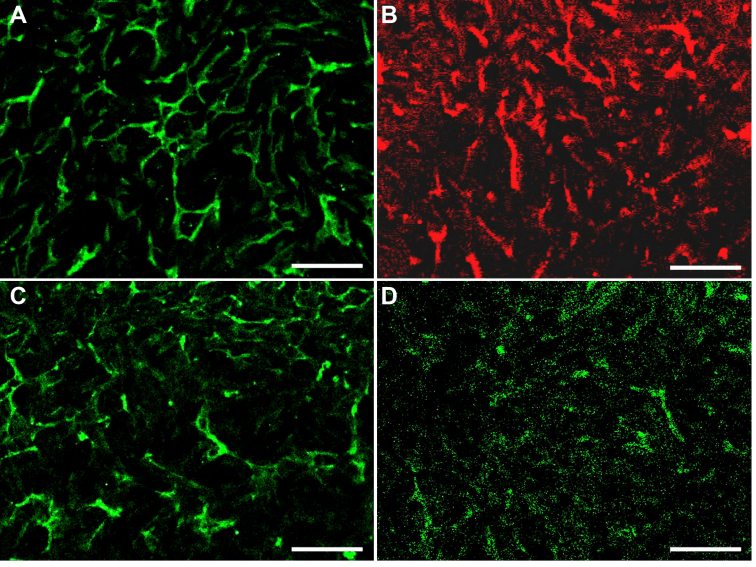
Immunofluorescence staining patterns of cell-junction proteins of HCE cells at passage 101. **A**: human zonula occludens protein 1 (ZO-1). **B**: human N-cadherin. **C**: human connecxin-43. **D**: human integrin αv/β5. Positive expression of the cell-junction proteins of HCE cells was shown. Scale bar: 50 μm.

Immunofluorescence staining pattern of cell-junction proteins are shown in Figure 6. Positive expression of ZO-1, N-cadherin, connexin 43, and integrin αv/β5 was detected in passage 101 HCE cells, implying that the cells still had potential of forming intercellular and cell-extracellular matrix (ECM) junctions.

### Gene expression of membrane transport proteins in HCE cells

**Figure 7:**
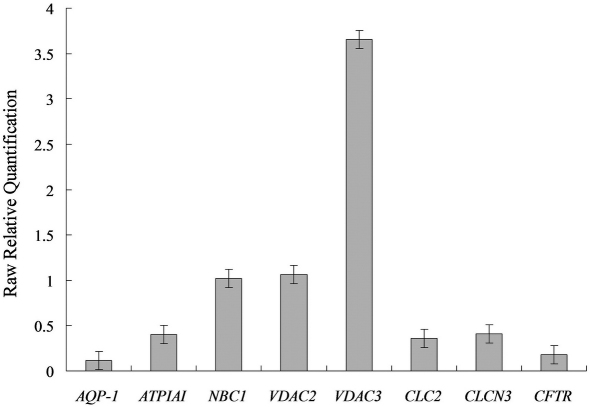
Real time PCR detection of the gene expression of membrane transport proteins of HCE cells at passage 101. Positive gene expression patterns of various membrane transport proteins of HCE cells were shown. *AQP1*, human aquaporin 1 gene; *ATP1A1*, human α1 polypeptide of Na^+^/K^+^-ATPase gene; *VDAC*, human voltage-dependent anion channel gene; *CLC*, human chloride channel protein gene; *NBC1*, human sodium bicarbonate cotransporter 4 gene; *CFTR*, human cystic fibrosis transmembrane conductance regulator gene. The housekeeping gene, human glyceraldehyde-3-phosphate dehydrogenase (*GAPDH*), was used as an internal control.

Results of real-time PCR revealed that the HCE cells at passage 101 still maintained the expression of various membrane transport protein genes such as *AQP1*, *ATP1A1*, *VDAC2*, *VDAC3*, *ClC2*, *ClC3*, *NBC1*, and *CFTR* (Figure 7), implying that the cells of the established cell line had properties of HCE cells and might have potentials to carry out membrane transport functions.

### Tumorigenicity of HCE cells

The results of tumorigenesis assay were shown in Table 2. Solid tumors were found in all 10 BalB/c nude mice 7 days after inoculated with HeLa cells, and grew progressively thereafter. However, no solid tumor was found in 10 BalB/c nude mice 60 days after passage 101 HCE cells were inoculated. The results indicated that the established HCE cell line had no tumorigenic potency.

**Table 2:** Tumorigenesis assay of passage 101 HCE cells in BALB/C nude mice.

**Inoculated cells**	**Total dose (cell/mouse)**	**Number of mice (% mortality)**	**Number of mice with tumor**
HCE cells	1.0×10^7^	10 (0)	0
HeLa cells	1.0×10^7^	10 (0)	10

Note: tumors were identified surgically.

### Biocompatibility of HCE cells to dAMs

**Figure 8:**
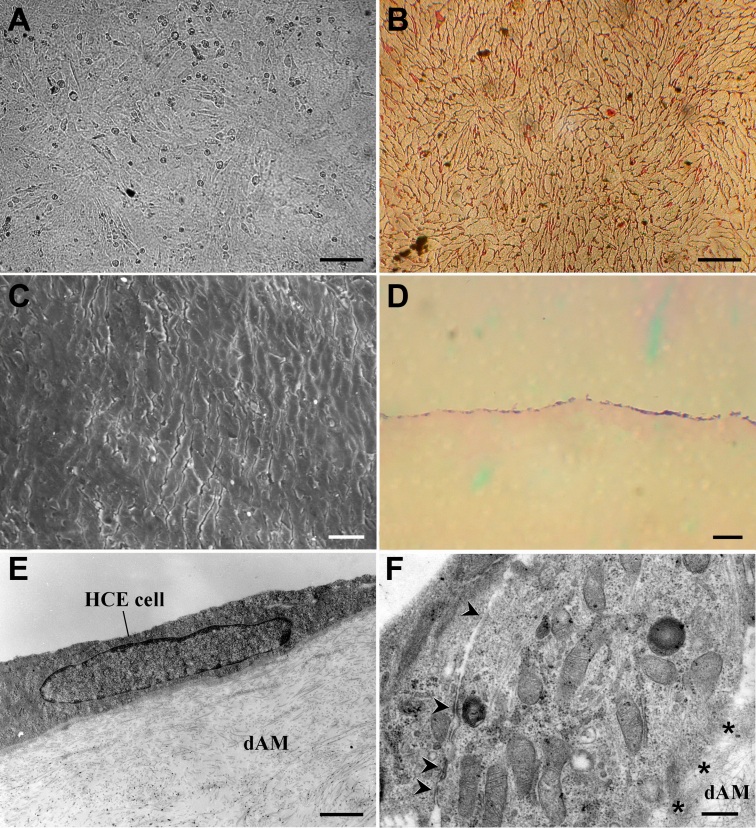
Evaluation of biocompatibility of passage 101 HCE cells on dAMs. **A**: The confluent cell sheet formed by HCE cells on dAM 116 h after inoculation. The cell density and elongated polygonal morphology were shown. Scale bar: 25 μm. **B**: alizarin red staining pattern of the HCE cell sheet. The cell density, elongated polygonal morphology and established intercellular junctions were shown. Scale bar: 25 μm. **C**: SEM image of the HCE cell sheet. The confluent cell sheet and elongated polygonal morphology of HCE cells were shown. Scale bar: 25 μm. **D**: hematoxylin-eosin (HE) staining pattern of the frozen section of the HCE cell sheet. The confluent monolayer cell sheet formed by HCE cells on dAM was shown. Scale bar: 100 μm. **E**: TEM image of the HCE cell sheet. The tightly attaching status of the cell sheet and dAM was shown. Scale bar: 4 μm. **F**: TEM image of the HCE cell sheet. The ultrastructure of HCE cells, the established cell-cell junctions (arrow heads) and cell-dAM junctions (asterisk) were shown. Scale bar: 500 nm.

**Figure 9:**
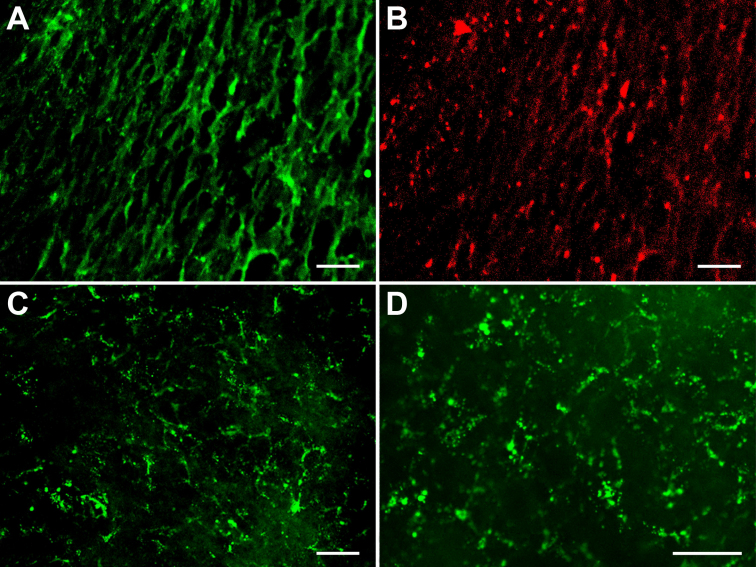
Immunofluorescence staining patterns of cell-junction proteins of the HCE cell sheet formed on dAM. **A**: human zonula occludens protein 1 (ZO-1). **B**: human N-cadherin. **C**: human connexin-43. **D**: human integrin αv/β5. Positive expression of the cell-junction proteins of passage 101 HCE cells on dAM was shown. Scale bar: 50 μm.

Passage 101 HCE cells grew very well on dAMs and formed confluent monolayer 116 h later with an average density of 3,413±111 cells/mm^2^ (Figure 8A). Most of the HCE cells were in slightly elongated polygonal morphology (Figure 8B,C), and the cell sheets they formed were continuous and intact that attached tightly to dAM (Figure 8D,E). The HCE cells of the cell sheets established various cell-cell and cell-dAM junctions (Figure 8F). These data suggested that the established HCE cell line had excellent biocompatibility to dAM, and could form confluent cell sheets on dAMs with various intercellular and cell-dAM junctions. Positive expressions of ZO-1, N-cadherin, connexin 43, and integrin αv/β5 were also detected in the HCE cells on dAM (Figure 9). All these indicated that the HCE cells had excellent biocompatibility to dAM, and the cells and dAM might be used for in vitro reconstruction of TE-HCE.

## Discussion

HCE cell lines are necessary in studies of HCE cell proliferation, differentiation, apoptosis, and TE-HCE reconstruction [16,17,20]. Even though numerous attempts have been made to obtain HCE cells in long-term culture, two main difficulties need to be surmounted in initiation of in vitro culture and successful subculture of the cells [7,8,10-15]. One is the difficulty in getting pure HCE cells, and the other is the difficulty in inducing HCE cells to divide in vitro [16,17]. Based on our previous experiences in rabbit corneal endothelial cell line establishment, manipulations of strictly controlled trypsin digestion, direct corneal fragment attaching, and successive detaching-reattaching of different durations were also used to obtain pure and enough HCE cells for primary culture initiation in the present study [16,17]. In addition, proliferation of HCE cells has been induced successfully in the present study by replenishing the culture medium with various supplements, such as chondroitin sulfate, glucosamine hydrochloride, N-acetylglucosamine hydrochloride, growth factors, ocular extracts, and culture supernatant of corneal stromal cells. The successful induction of proliferation of HCE cells was consistent with the results obtained in cultured HCE cells [21,22] and rabbit corneal endothelial cells [16,17].

With pure HCE cells and supplements-induced proliferation, a continuous untransfected HCE cell line, with the modal chromosome number of 46, had been successfully established and subcultured to passage 224 in this study. The cells proliferated actively and constantly with a population doubling time of 26.2 h at passage 101. The estimated cell doubling time was faster than that of a previously established immortalized HCE cell line (30.15±10.96 h) [15].

To characterize corneal endothelial cells, various marker proteins such as NSE (a neuroectodermal origin marker protein of HCE cells), collagen type IV (a marker protein of HCE cells), FLK1 (VEGF receptor 2, a marker protein of both vascular and corneal endothelial cells), vWF (a marker protein of vascular endothelial cells), and keratin-12 (a marker protein of corneal epithelial cells) have often been used [14,16,23-26] and were also employed in this study. Positive expression of NSE, collagen type IV and FLK1, but not vWF and keratin 12, combined with the results of morphology recovery suggests that the HCE cell line established in this study was of a corneal endothelial origin, consistent with the results obtained in previous studies of HCE cells [13,27] and rabbit corneal endothelial cells [16,17]. Taken together, these results indicated that established cell line was a pure HCE cell line without contamination of corneal epithelial and stromal cells.

Since cell junctions are crucial for HCE cells to maintain intact endothelium for achieving stable corneal hydration status, determination of the expression of junction proteins was vital in HCE cell characterization [16]. HCE cells have been found to have tight junction proteins, gap junction proteins, and anchoring junction proteins, which mediate and strengthen cell-cell and cell-matrix associations [16,28-30]. In this study, HCE cells at passage 101 still maintained stable expression of ZO-1 (an intercellular tight junction protein), connexin-43 (an intercellular gap junction protein), N-cadherin (an intercellular anchoring junction protein), and integrin αv/β5 (a cell-matrix anchoring junction protein), suggesting that the HCE cells still maintained potency to establish normal cell-cell and cell-ECM junctions. This expression pattern of cell junction proteins in HCE cells is consistent with the results obtained from rabbit corneal endothelial cells [16], but is different from that of in vitro cultured HCE cells [31].

Membrane transport proteins of HCE cells, such as AQP1, Na^+^/K^+^-ATPase, VDACs, CLCs, NBC1 and CFTR, play crucial roles in maintaining corneal dehydration and transparency [16,32-34]. Among these, AQP1 is important for osmotically driven water transport [35], and Na^+^/K^+^-ATPase for maintaining corneal dehydration and transparency [36], VDACs for anion transport [37,38], CLCs for chloride transport [39,40], NBC1 for the cotransport of Na^+^ and HCO_3_^-^ [41], and CFTR for HCO_3_^-^ transport [42,43]. In the present study, the maintenance of gene expressions of *AQP1*, *ATP1A1*, *VDAC2*, *VDAC3*, *CLC2*, *CLC3*, *NBC1*, and *CFTR*, indicated that the established HCE cell line still had potencies to carry out normal functions of transmembrane transport, consistent with the results obtained from rabbit corneal endothelial cell lines [16].

Immortalized corneal endothelial cell lines by oncogene transfections may have latent potency of tumorigenicity and abnormal phenotypes, and cannot be used in studies of HCE cells and reconstruction of the ocular surface with a tissue-engineered cornea [16,17]. Tumorigenic potency assay with nude mice in this study showed that the HCE cell line had no latent potency for tumorigenicity, suggesting that the HCE cell line established in this study could be safely used in studies of HCE cells and in vitro reconstruction of TE-HCEs.

Excellent biocompatibility between corneal cells and scaffold carriers is a vital precondition for in vitro reconstruction of tissue-engineered corneas [16,44]. Among the scaffold carriers used, dAM has been proving to have excellent biocompatibility to HCE cells and successfully used in TE-HCE reconstruction [26,27]. In the present study, the cells from established HCE cell line formed confluent cell sheets on dAMs with various intercellular and cell-dAM junctions. And the average cell density of the HCE cell sheets on dAMs was about 3,413±111 cells/mm^2^, similar to that of a 10–11 years old child [45], which was much higher than those of previous reports [26,27,46]. These results indicate that the HCE cells could form integral endothelium-like structures on dAMs, consistent with the results obtained from passage 5 HCE cells and immortalized HCE cells on dAMs [26,27]. The excellent biocompatibility to dAMs and high cell density of reconstructed cell sheets imply that the established HCE cell line might be feasible for reconstruction studies of TE-HCE [47].

In conclusion, a continuous untransfected and non-tumorigenic HCE cell line, with normal protein expression and excellent biocompatibility to dAMs, has been established successfully in this study. And the cell line provides a powerful tool for studies of proliferation, differentiation and apoptosis of HCE cells. It could also be used for reconstruction of TE-HCE, providing a promising method for the treatment of diseases caused by corneal endothelial disorders. Studies on TE-HCE reconstruction and its animal transplantation are currently underway in our laboratory.
